# Effect of 10-year cumulative blood pressure exposure on atherosclerotic cardiovascular disease of different age groups: kailuan cohort study

**DOI:** 10.3389/fcvm.2024.1341097

**Published:** 2024-02-01

**Authors:** Lu Guo, Faming Tian, Jingyao Wang, Wenqi Xu, Wenjuan Li, Xiaoli Hou, Mengyi Zheng, Xuemei Yang, Lishu Gao, Shuohua Chen, Nan Zhang, Shouling Wu

**Affiliations:** ^1^ The School of Public Health, North China University of Science and Technology, Tangshan, Hebei, China; ^2^ The School of Clinical Medicine, North China University of Science and Technology, Tangshan, Hebei, China; ^3^ Cardiovascular Center, Beijing Tongren Hospital, Capital Medical University, Beijing, China; ^4^ Department of Endocrinology, Tangshan People’s Hospital, Tangshan, Hebei, China; ^5^ Department of Cardiology, Kailuan General Hospital, Tangshan, Hebei, China; ^6^ Department of Orthopedics, Kailuan General Hospital, Tangshan, Hebei, China

**Keywords:** time-weighted average blood pressure, cumulative blood pressure, atherosclerotic cardiovascular disease, different ages, cohort study

## Abstract

**Background:**

The level at which cumulative blood pressure (BP) can increase the risk of ASCVD in different age groups remains unclear. This study aimed to investigate the association of 10-year cumulative BP levels with the long-term risk of ASCVD of different age groups.

**Methods:**

Cumulative BP exposure was assessed using the time-weighted average (TWA) BP divided into four BP groups. The participants were also divided into four groups according to their baseline age (<50, 50–59, 60–69, or ≥70 years). The association between TWA BP and the risk of ASCVD was assessed by age group using multivariate Cox models. The China-PAR prediction model was used to assess the ability of TWA BP to predict ASCVD.

**Results:**

In the group aged <50 years, the hazard ratios and 95% confidence intervals for the risk of ASCVD were 2.66 (1.04–6.80), 3.38 (1.54–7.43), and 3.13 (1.36–7.24) for the elevated BP, stage 1 hypertension, and stage 2 hypertension groups, respectively, when compared with the normal BP group. There was a significant difference in the risk of ASCVD between the age groups, with participants aged <50 years having the highest risk, followed by those aged 50–59, 60–69, and ≥70 years.

**Conclusions:**

The risk of ASCVD with high cumulative BP exposure was age-dependent, with a gradual decrease in risk with increasing age.

## Introduction

Hypertension is a common chronic disease and one of the major risk factors for atherosclerotic cardiovascular disease (ASCVD) (1). According to the most recent statistics (2), the number of hypertensive patients in China has reached 245 million, the prevalence of hypertension among adults aged 18 and above to be 27.9%, which is increasing in incidence and prevalence year by year. A study by Wang et al (3) found that 56.4% of strokes and 54.6% of coronary events were caused by hypertension. A study by Lewington et al (4) found that for blood pressure (BP) from 115/75 mmHg onwards (1 mmHg = 0.133 kPa), each 20/10-mmHg increment is associated with a 2-fold increase in the risk of ASCVD. More recent studies have found that the association between new-onset hypertension and cardiovascular risk differs according to age group (5, 6). Furthermore, a prospective study noted that the risk of cardiovascular events decreased with increasing age of onset of hypertension (7). That study also found that the risk of cardiovascular events from early BP exposure in young people is greater than that from late exposure.

Previous estimates of the risk of ASCVD have been limited to a single or short period of BP measurement (7–9). However, there are data suggesting that cumulative blood pressure (CumBP), unlike a single BP measurement, better reflects the effect of long-term changes in BP on ASCVD over time (10, 11). However, there is limited information on the effect of CumBP on the risk of ASCVD over time. Moreover, there are no studies on the risk of ASCVD in different age groups as a result of CumBP exposure. Although studies by Wang et al (12) and Reges et al (10) explored the relationship between CumBP and cardiovascular disease (CVD) in different age groups, both these studies had the limitations of small sample sizes in each age group after stratification and a small number of CumBP measurements. The aim of this study was to determine and compare the risk and distribution of CumBP exposure and the development of ASCVD in specific age groups by analyzing the effect of 10 years of CumBP exposure on ASCVD at different ages using the KaiLuan study cohort (registration number: ChiCTR-TNC-11001489), which includes up to 15 years of longitudinal data.

## Methods

### Study design and participants

From 2006 to 2007, employees and retirees of the Kailuan Group at the age of 18–98 were recruited to participate in the the first health checkups and repeated the checks every 2 years, obtaining data including systolic blood pressure (SBP) and diastolic blood pressure (DBP) as well as follow-up information. A detailed description of the study design has been published elsewhere (13). We have completed seven physical examinations to date, and the occurrence of ASCVD in the observed subjects is recorded annually by Kailuan General Hospital and its affiliated hospitals.

Individuals were included in the present study based on the following inclusion

criteria and data requirements: individuals who attended the health checkups from 2006 to 2018 were included in this study for calculation of the 10-year CumBP. In the present study, a total of 64,642 individuals from the Kailuan Group who participated in health checkups in 2006 and 2016 or in 2008 and 2018 and attended at least one physical examination during two annual visits (2006–2016 and 2008–2018) were included. Index date was defined as the earliest exam that inclusion criteria are met and the baseline was established at year 10. We excluded the following: 3,510 participants that with missing BP data, 5,682 participants with previous ASCVD at baseline (myocardial infarction [MI], including hemodynamic reconstruction, heart failure [HF], and ischemic stroke), a total of 55,450 participants were enrolled in the final analysis (Supplementary Figure S1). The study was approved by the ethics committee of Kailuan General Hospital and conducted in accordance with the Declaration of Helsinki. All participants provided written informed consent to be included in the study.

### Data collection

Information on demographic and lifestyle factors, medical history, and medication history was collected by an structured questionnaire and confirmed item-by-item during face-to-face interviews conducted by trained medical and nursing staff, as described elsewhere (14, 15). The portable stadiometers were used to measure height, and calibrated platform scales were used for measuring body weight. Both the weight and height of all the participants were measured without heavy clothing or any other accessories, for example, shoes and hats. BMI was calculated as weight (kg)/height^2^ (m^2^). Fasting blood sample (8–10 h) of 5 ml was taken from the anterior elbow vein on an empty stomach from 7 to 9 am, and the blood was injected into a vacuum tube containing EDTA. The supernatant was centrifuged at room temperature and measured within 4 h. Fasting blood glucose (FBG) was measured using the hexokinase/glucose-6- phosphate dehydrogenase method (Mind Bioengineering Co. Ltd, Shanghai, China). Using the same blood sample, concentrations of triglycerides, low-density lipoprotein cholesterol (LDL-C), and high density lipoprotein cholesterol (HDL-C) were measured by auto-analyzer (Hitachi 747, Tokyo, Japan) at the central laboratory of Kailuan Hospital. The specific methods used for determination of the assay indicators have been reported elsewhere (16). Covariates definitions was shown in Supplementary Table S1.

### Assessment of BP

BP was measured between 7:00 a.m. and 9:00 a.m. on the examination day. Participants had rested in a seated position without smoking or consuming tea or coffee for 15 min before measurements were obtained. BP was measured in the right brachial artery by a trained and qualified medical staff member using a calibrated a manual sphygmomanometer. SBP and DBP were recorded during auscultation of the first and fifth Korotkov sounds. The average of three consecutive measurements taken at 1-min or 2-min intervals was recorded.

### Calculation of time-weighted average BP

CumBP exposure was calculated as the time-weighted average (TWA) BP using measurements obtained at the time of the physical examination between 2006 and 2007 and 2016–2017 or between 2008 and 2009 and 2018–2019. TWA BP was calculated as the average of two consecutive SBP and DBP measurements multiplied by the sum of the corresponding time intervals divided by the corresponding interval. For detailed calculation of TWA BP, see Supplementary Table S2.

### BP groups

In accordance with the 2017 American College of Cardiology/American Heart Association (ACC/AHA) Guidelines for the Prevention, Detection, Evaluation, and Management of Hypertension in Adults (17), the calculated baseline TWA BP was divided into four groups: normal BP (SBP <120 mmHg/DBP <80 mmHg); elevated BP (SBP 121–129 mmHg/DBP < 80 mmHg); stage 1 hypertension (SBP 130–139 mmHg or DBP 80–89 mmHg); and stage 2 hypertension (SBP ≥140 mmHg, DBP >90 mmHg, taking antihypertensive medication, or a history of hypertension; According to current treat guidelines, stage 1 hypertension is not required for initiation of antihypertensive medication treatment.Therefore, taking antihypertensive medications were included in the stage 2 hypertension group).

### Age groups

The study participants were divided into four age groups based on whether they were aged <50 years, 50–59 years, 60–69 years, or ≥70 years at baseline.

### Study outcomes and follow-up

**Figure 1:**
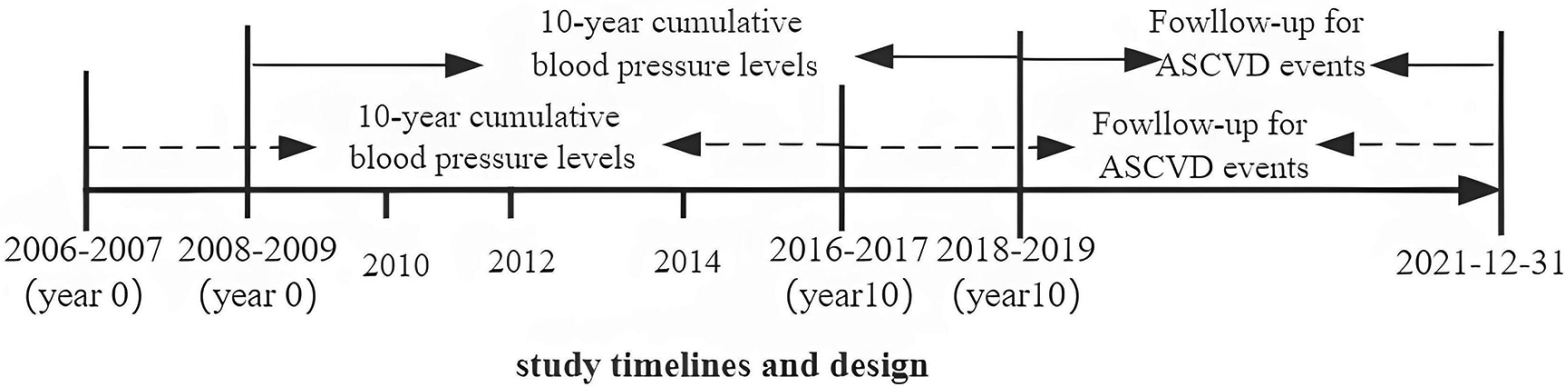
Study timelines and design.

The date of the final physical examination (the final year of the 10-year window) was taken as the starting point for follow-up. Each participant's observation time ended at the time of an incident ASCVD event (i.e., MI, HF, or ischemic stroke) or on December 31, 2021; two or more ASCVD events are counted as one ASCVD event based on the time of the first event. ASCVD was defined as coronary heart disease or nonfatal MI, fatal or nonfatal ischemic stroke, or HF. Physician adjudicators made the diagnosis based on World Health Organization criteria, clinical symptoms, computed tomography scans, magnetic resonance images, electrocardiograms, and other diagnostic reports or based on the European Society of Cardiology guidelines (18–21). The timeline and design of the study are shown in Figure 1.

### Statistical analysis

Continuous data that were distributed normally are shown as the mean ± standard deviation. Multiple groups were compared using analysis of variance. Parameters with a skewed distribution are expressed as the median and interquartile range. Groups were compared using the Wilcoxon signed-rank test. Categorical data are expressed as the frequency and percentage and were compared between groups using the chi-squared test. The 10-year TWA BP values were calculated and grouped using the SAS program. The cumulative incidence of ASCVD according to TWA BP was calculated in the different age groups based on Kaplan-Meier methods, and differences in cumulative incidence were examined using the log-rank test. Sex-standardization absolute risk ratios of different time-averaged BP categories at different age groups were estimated using an absolute risk regression model. A Cox proportional hazards regression model was constructed based on Schoenfeld residuals to calculate hazard ratios (HRs) and 95% confidence intervals (CIs) for ASCVD in the TWA BP groups. Multivariate analyses were based on the following: sex, smoking, drinking, education level, physical activity, BMI, triglycerides, LDL-C, HDL-C, FBG, steatotic liver disease, and glucose-lowering medication (model 1); model 1 + antihypertensive medication (model 2); model 2 + lipid-lowering medication (model 3); and model 3 + SBP at baseline (model 4). To test the robustness of the results, we repeated the Cox model and performed two sensitivity analysis: (1) After excluding the population that did not complete six consecutive health checkups. (2) Regrouping the stage 1 hypertension and stage 2 hypertension groups. (participants who were taking anti-hypertensive medications and had blood pressure levels of 130 mmHg ≤ Time-averaged SBP < 140 mmHg or 80 mmHg ≤ Time-averaged DBP < 90 mmHg or less were classified as stage 1 hypertension).

The concordance index (C-index), integrated discrimination improvement (IDI), and net reclassification index (NRI) were calculated using the China-PAR prediction model to evaluate the ability of TWA SBP and TWA DBP to predict ASCVD. In addition, the predictive values of TWA BP determinations for ASCVD occurrence was evaluated by the area under curve (AUC), which was calculated from the receiver operating characteristic (ROC) curve analyses. The statistical analysis was performed using SAS version 9.4 software (SAS Institute Inc., Cary, NC, USA). A *P*-value < 0.05 was considered statistically significant.

## Results

### Baseline characteristics

A total of 55,450 study participants were included in the present analysis. The mean age was 57.24 ± 11.43 years and 42,201 (76.11%) of the participants were male. The mean number of BP measurements was 5.38 ± 0.83. There were 10,535 participants in the normal BP group, 4,343 in the elevated BP group, 8,382 in the stage 1 hypertension group, and 32,190 in the stage 2 hypertension group (Table 1); the 10-year TWA SBP was 112.31 ± 5.45, 123.70 ± 2.65, 127.48 ± 5.84, and 140.76 ± 14.25 mmHg, respectively, and the 10-year TWA DBP was 73.23 ± 4.09, 76.87 ± 2.62, 82.73 ± 2.96, and 87.69 ± 8.19 mmHg, respectively, for the four hypertension groups.

**Table 1:** Characteristics of the participants according to time-averaged BP categories.

Variables	Total	Normal BP	Elevated blood pressure	Stage 1 hypertension	Stage 2 hypertension	*P*-Value
	(*N* = 55,450)	(*N* = 10,535)	(*N* = 4,343)	(*N* = 8,382)	(*N* = 32,190)	
Age, year	57.24 ± 11.43	51.01 ± 11.00	55.85 ± 12.26	55.62 ± 11.22	59.88 ± 10.58	<0.001
Male, *N* (%)	42,201 (76.11)	5,948 (56.46)	3,238 (74.56)	6,955 (82.98)	26,060 (80.96)	<0.001
Smoker, *N* (%)	17,058 (30.76)	2,750 (26.10)	1,344 (30.95)	2,722 (32.47)	10,242 (31.82)	<0.001
Drinker, *N* (%)	16,433 (29.64)	2,465 (23.40)	1,114 (25.65)	2,560 (30.54)	10,294 (31.98)	<0.001
≥Senior high school, *N* (%)	14,322 (25.83)	4,356 (41.35)	1,294 (29.80)	2,405 (28.69)	6,267 (19.47)	<0.001
Physical exercise, *N* (%)	6,300 (11.36)	996 (9.45)	488 (11.24)	789 (9.41)	4,027 (12.51)	<0.001
BMI, kg/m^2^	25.11 ± 3.39	23.82 ± 3.22	24.57 ± 3.25	25.10 ± 3.28	25.61 ± 3.37	<0.001
SBP mmHg	138.15 ± 19.58	118.19 ± 10.05	126.80 ± 8.31	127.74 ± 8.18	148.93 ± 17.56	<0.001
DBP mmHg	81.52 ± 10.98	72.39 ± 7.60	74.65 ± 7.29	79.40 ± 6.59	85.98 ± 10.75	<0.001
Time-averaged SBP mmHg	132.01 ± 15.99	112.31 ± 5.45	123.70 ± 2.65	127.48 ± 5.84	140.76 ± 14.25	<0.001
Time-averaged DBP mmHg	83.35 ± 8.82	73.23 ± 4.09	76.86 ± 2.62	82.73 ± 2.96	87.69 ± 8.19	<0.001
WC cm	87.82 ± 9.22	83.90 ± 8.89	86.77 ± 8.62	88.29 ± 8.84	89.12 ± 9.13	<0.001
TC mmol/L	4.72 ± 1.67	4.61 ± 1.65	4.61 ± 1.61	4.71 ± 1.62	4.77 ± 1.69	<0.001
TG mmol/L M (P25,P75)	1.47 (0.98,2.55)	1.23 (0.84,2.03)	1.37 (0.93,2.34)	1.45 (0.98,2.42)	1.59 (1.06,2.78)	<0.001
HDL_C mmol/L	1.51 ± 0.52	1.55 ± 0.51	1.49 ± 0.45	1.46 ± 0.51	1.51 ± 0.54	<0.001
LDL_C mmol/L	2.95 ± 0.85	2.86 ± 0.80	2.94 ± 0.79	2.92 ± 0.86	2.99 ± 0.87	<0.001
FBG mmol/L	5.90 ± 1.90	5.34 ± 1.24	5.63 ± 1.51	5.71 ± 1.65	6.18 ± 2.13	<0.001
Antihypertensive treatment, *N* (%)	16,725 (30.16)	_	_	_	16,725 (51.96)	<0.001
Lipid-lowering treatment, *N* (%)	4,371 (7.88)	286 (2.71)	177 (4.08)	364 (4.34)	3,544 (11.01)	<0.001
Antidiabetic treatment, *N* (%)	3,836 (6.92)	236 (2.24)	195 (4.49)	365 (4.35)	3,040 (9.44)	<0.001
Diabetes, *N* (%)	8,777 (15.83)	582 (5.52)	447 (10.29)	978 (11.67)	6,770 (21.03)	<0.001
Steatotic liver disease, *N* (%)	24,858 (44.83)	3,068 (29.12)	1,696 (39.05)	3,704 (44.19)	16,390 (50.92)	<0.001

SBP, systolic blood pressure; DBP, diastolic blood pressure; BMI, body mass index; WC, waist circumference; FBG, fasting blood glucose; LDL-C, low-density lipoprotein cholesterol; HDL-C, high-density lipoprotein cholesterol; TG, triglyceride; TC, total cholesterol.

Data are presented as *n* (%), mean ± SD or median (interquartile range) [M (P25, P75 )] according to variable category.

*P*, comparison of baseline characteristics among different BP groups.

Compared with the normal BP group, the elevated BP group, stage 1 hypertension group, and stage 2 hypertension group were prone to be older, had much higher BMI, baseline BP, 10-year TWA SBP, 10-year TWA DBP, TG, FBG, LDL-C, HDL-C, and had higher prevalences of smoking, drinking, taking anti-hypertensive, hypoglycemic, or lipid-lowering medications, diabetes, and steatotic liver disease (all *P *< 0.05; Table 1) at baseline examination.

### Incidence of ASCVD according to CumBP exposure

During a mean follow-up of 3.81 ± 1.00 years, we observed 2,197 (3.96%) incident ASCVD, including 1,357 (2.45%) with stroke, 698 (1.73%) with MI, and 235 (0.58%) with HF. The number of cases of ASCVD in each of the four hypertension groups was 104 (0.99%), 105 (2.42%), 227 (2.71%), and 1,761 (5.47%), respectively. In total, 137 (0.96%) of the ASCVD cases were aged <50 years, 600 (3.69%) were aged 50–59 years, 946 (5.07%) were aged 60–69 years, and 514 (8.30%) were aged ≥70 years. There were an significant differences among the groups in the rate of adverse outcomes (Supplementary Table S3). Kaplan-Meier analysis estimated the 4-year cumulative incidence of ASCVD in each of the four hypertension groups was 1.04%, 2.58%, 2.73%, and 8.72%, respectively (Supplementary Figure S2);. The cumulative incidence of ASCVD increased with time in all four CumBP exposure groups in different age groups, and all differences were statistically significant (*P *< 0.05, log-rank test; Supplementary Figure S3). The respective sex-standardization ASCVD incidence absolute risk in the four TWA BP groups were 0.19%, 0.62%, 0.96%, and 1.73%, 1.09%, 2.31%, 2.43%, and 5.00%, 1.92%, 2.96%, 3.18%, and 6.11%, and 6.17%, 6.15%, 5.70%, and 9.04% (Supplementary Table S4).

### Cox proportional risk model for risk of ASCVD according to CumBP exposure and age group

The risk models for the different BP groups and ASCVD were established by multivariate Cox regression model analysis. In the population aged <50 years, compared with the normal BP group, the HRs and 95% CIs for the risk of ASCVD were 2.66 (1.04–6.80), 3.38 (1.54–7.43), and 3.13 (1.36–7.24), respectively, for the elevated BP, stage 1 hypertension, and stage 2 hypertension groups. The HRs and 95% CIs for ASCVD were 1.45 (1.12–1.87) and 1.44 (1.18–1.74) per standard deviation increase in CumSBP and CumDBP, respectively. In the population aged 50–59 years, the HRs and 95% CIs for the risk of ASCVD in the elevated BP, stage 1 hypertension, and stage 2 hypertension groups were 1.82 (1.09–3.04), 1.72 (1.12–2.64), and 2.32 (1.55–3.47), respectively, when compared with the normal BP group. The HRs and 95% CIs for ASCVD were 1.26 (1.12–1.41) and 1.22 (1.11–1.34) per standard deviation increase in CumSBP and CumDBP, respectively. In the population aged 60–69 years, the HRs and 95% CIs for the risk of ASCVD in the elevated BP, stage 1 hypertension, and stage 2 hypertension groups were 1.28 (0.83–1.96), 1.58 (1.10–2.28), and 1.62 (1.14–2.29), respectively, when compared with the normal BP group. The HRs and 95% CIs for ASCVD were 1.13 (1.03–1.23) and 1.08 (1.00–1.16) per standard deviation increase in CumSBP and CumDBP, respectively. In the population aged ≥70 years, the HRs and 95% CIs for the risk of ASCVD in the elevated BP, stage 1 hypertension, and stage 1 hypertension groups were 1.08 (0.61–1.92), 1.04 (0.61–1.78), and 1.21 (0.75–1.96) when compared with the normal BP group. The HRs and 95% CIs for ASCVD were 1.06 (0.95–1.18) and 0.98 (0.89–1.08) per standard deviation increase in CumSBP and CumDBP, respectively (Table 2). We repeated the Cox model after excluding study participants who did not complete six consecutive health checkups (Supplementary Table S5), and regrouping according to take anti-hypertensive medications (Supplementary Table S6). And found that the risk of ASCVD in the different BP and age groups was generally consistent with the main statistical analysis.

**Table 2:** Risk of occurrence of atherosclerotic cardiovascular disease events estimated based on time-averaged BP at different ages.

Age categories	Cumulative BP categories (mmHg)	Cases/total (*N*)	Incidence density (per 1,000 person-years)	HR (95%CI)			
				Model 1	Model 2	Model 3	Model 4
<50 years	Normal BP	9/4,953	0.47	Reference	Reference	Reference	Reference
	Elevated blood pressure	9/1,356	1.74	2.90 (1.14–7.41)	2.93 (1.15–7.47)	2.87 (1.13–7.32)	2.66 (1.04–6.80)
	Stage 1 hypertension	25/2,587	2.52	3.81 (1.75–8.33)	3.85 (1.76–8.43)	3.64 (1.67–7.96)	3.38 (1.54–7.43)
	Stage 2 hypertension	94/5,442	4.44	5.96 (2.92–12.16)	4.20 (1.91–9.25)	3.92 (1.78–8.63)	3.13 (1.36–7.24)
	Per 1 SD (10yTWA-SBP)			1.84 (1.55–2.20)	1.66 (1.35–2.03)	1.53 (1.24–1.90)	1.45 (1.12–1.87)
	Per 1 SD (10yTWA-DBP)			1.73 (1.50–1.99)	1.60 (1.36–1.89)	1.49 (1.26–1.77)	1.44 (1.18–1.74)
50–59 years	Normal BP	33/3,068	2.77	Reference	Reference	Reference	Reference
	Elevated blood pressure	27/1,125	6.09	1.95 (1.17–3.25)	1.95 (1.17–3.25)	1.99 (1.19–3.31)	1.82 (1.09–3.04)
	Stage 1 hypertension	60/2,491	6.18	1.87 (1.22–2.87)	1.87 (1.22–2.87)	1.89 (1.23–2.90)	1.72 (1.12–2.64)
	Stage 2 hypertension	480/9,561	13.14	3.69 (2.58–5.29)	3.49 (2.39–5.09)	3.31 (2.26–4.83)	2.32 (1.55–3.47)
	Per 1 SD (10yTWA-SBP)			1.59 (1.46–1.72)	1.54 (1.41–1.68)	1.44 (1.31-.1.57)	1.26 (1.12–1.41)
	Per 1 SD (10yTWA-DBP)			1.47 (1.37–1.58)	1.42 (1.31–1.54)	1.34 (1.24–1.46)	1.22 (1.11–1.34)
60–69 years	Normal BP	41/2,142	4.97	Reference	Reference	Reference	Reference
	Elevated blood pressure	43/1,439	7.80	1.48 (0.96–2.27)	1.48 (0.97–2.27)	1.36 (0.89–2.09)	1.28 (0.83–1.96)
	Stage 1 hypertension	104/2,660	10.27	1.79 (1.24–2.57)	1.79 (1.25–2.58)	1.70 (1.18–2.44)	1.58 (1.10–2.28)
	Stage 2 hypertension	758/12,436	16.29	2.67 (1.94–3.67)	2.44 (1.76–3.37)	2.14 (1.55–2.96)	1.62 (1.14–2.29)
	Per 1 SD (10yTWA-SBP)			1.33 (1.25–1.41)	1.29 (1.21–1.38)	1.24 (1.16–1.33)	1.13 (1.03–1.23)
	Per 1 SD (10yTWA-DBP)			1.23 (1.16–1.30)	1.18 (1.10–1.26)	1.16 (1.08–1.24)	1.08 (1.00–1.16)
70 ≥ years	Normal BP	21/372	14.98	Reference	Reference	Reference	Reference
	Elevated Blood Pressure	26/423	17.11	1.11 (0.63–1.98)	1.12 (0.63–1.99)	1.11 (0.63–1.98)	1.08 (0.61–1.92)
	Stage 1 Hypertension	38/644	16.31	1.06 (0.62–1.81)	1.06 (0.62–1.81)	1.08 (0.63–1.84)	1.04 (0.61–1.78)
	Stage 2 Hypertension	429/4,751	25.08	1.54 (0.99–2.39)	1.40 (0.89–2.20)	1.39 (0.88–1.18)	1.21 (0.75–1.96)
	Per 1 SD (10yTWA-SBP)			1.15 (1.06–1.24)	1.11 (1.02–1.21)	1.11 (1.01–1.21)	1.06 (0.95–1.18)
	Per 1 SD (10yTWA-DBP)			1.05 (0.96–1.15)	1.01 (0.92–1.11)	1.02 (0.93–1.12)	0.98 (0.89–1.08)

SBP, systolic blood pressure; DBP, diastolic blood pressure; SD, standard deviation; 10 y, ten years; TWA, time-weight average;.

Model 1: adjusted for sex, drinking, smoking, education level, BMI, physical exercise, LDL-C, HDL-C, TG, FBG, steatotic liver disease, and Antidiabetic treatment.

Model 2: adjusted for model 1 plus anti-hypertensive drug.

Model 3: adjusted for model 2 plus use of the Lipid-lowering drug.

Model 4: adjusted model 3 plus systolic blood pressure at baseline.

### Ability of TWA SBP and TWA DBP to predict ASCVD

**Figure 2:**
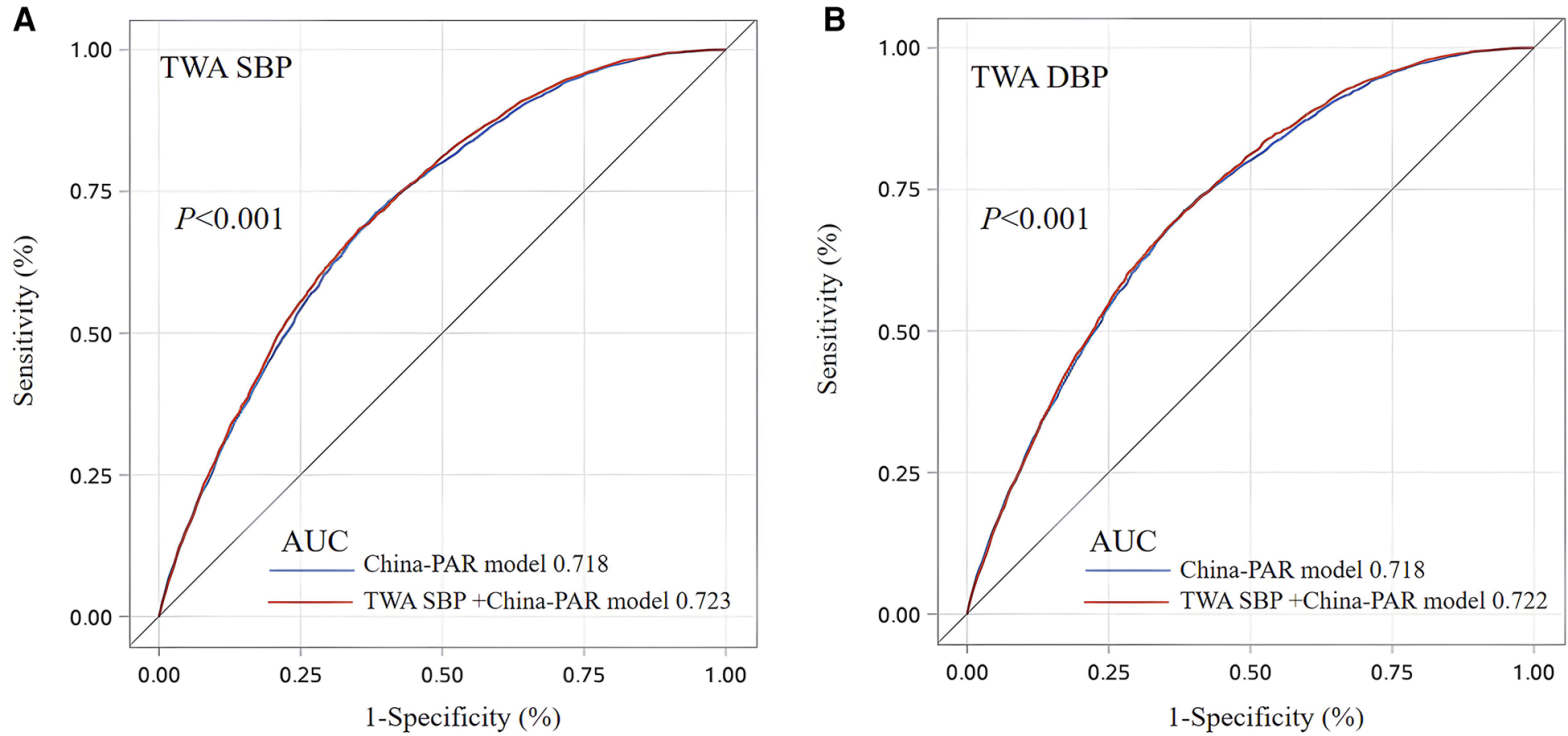
Receiver operating characteristic analysis for baseline BP and TWA BP distinguishing new ASCVD occurrence. TWA SBP (**A**) and TWA DBP (**B**) TWA, Time-Weight average, SBP, systolic blood pressure; DBP, diastolic blood pressure; AUC, area under curve. The best cutoff value represents the TWA SBP was 138.50 mmHg for predicting ASCVD with a sensitivity of 78.65% and a 1-specificity of 46.62%. the TWA DBP cutoff value was 88.68 mmHg for predicting ASCVD with a sensitivity of 67.91% and a 1-specificity of 34.97%. China-PAR model: ln(age) + ln(SBP) + ln(TC) + ln(HDL_C) + ln(WC)+smoke + diabetes + history of familial ASCVD + ln(age)*ln(SBP)+ln(age)*smoke + ln(age)*history of familial ASCVD.

The predictive value of the 10-year TWA BP was higher than that of the original prediction model for the risk of ASCVD. The C-index of TWA SBP and TWA DBP for prediction of ASCVD were 0.717, 0.714, respectively. Which were improved by 0.4% and 0.1% when compared with the C-index of the China-PAR prediction model (0.713). The NRI was 0.045 (*P *< 0.05) with an overall improvement in correct classification ability of 4.5%. The integrated discrimination improvement was 0.0003 (*P *= 0.132) and the CumSBP correct prediction probability improved by 0.03%. Although the C-index of TWA DBP improved, the IDI and NRI index did not change (Table 3). We compared the predictive values (AUC) of the baseline BP for ASCVD occurrence with the combined model of baseline BP plus TWA BP in China-PAR model, and found that the difference was statistically significant (all *P* < 0.001). The predictive ability (AUC) was increased only by 0.5% for TWA SBP and 0.4% for TWA DBP, respectively (Figure 2)**.**

**Table 3:** Predictive and discriminatory performance of blood pressure measures for atherosclerotic cardiovascular disease.

ASCVD events	C-index (95% CI)	Category-free NRI (SE)	IDI (SE)
China-PAR model	0.713 (0.703–0.723)	Reference	Reference
New model 1	0.717 (0.708–0.727)	0.045, *p = 0.040*	0.0003, *p = 0.132*
New model 2	0.714 (0.704–0.724)	0.028, *p *= 0.192	−0.0007, *p *= 0.012

China-PAR model: ln(age) + ln(SBP) + ln(TC) + ln(HDL_C) + ln(WC) + smoke + diabetes + history of familial ASCVD + ln(age)*ln(SBP) + ln(age)*smoke + ln(age)*history of familial ASCVD.

New model 1: Time-averaged SBP + ln(age) + ln(Time-averaged SBP) + ln(TC) + ln(HDL_C) + ln(WC) + smoke + diabetes + history of familial ASCVD + ln(age)*ln(Time-averaged SBP) + ln(age)*smoke + ln(age)*history of familial ASCVD.

New model 2: Time-averaged DBP + ln(age) + ln(Time-averaged DBP) + ln(TC) + ln(HDL_C) + ln(WC) + smoke + diabetes + history of familial ASCVD + ln(age)*ln(Time-averaged DBP) + ln(age)*smoke + ln(age)*history of familial ASCVD.

## Discussion

Our main finding in this study was that 10 years of high CumBP exposure was positively associated with a higher risk of ASCVD. Individuals exposed to high incremental BP levels early in life may have a higher risk of ASCVD. The risk of ASCVD with high CumBP exposure was age-dependent, with a gradual decrease in risk with increasing age. Thus, CumBP may have a higher predictive value than BP at baseline and improve the classification of risk of ASCVD.

In this study, we found that after correcting for confounders, including baseline SBP, the risk of ASCVD was increased according to CumBP in the elevated BP, stage 1 hypertension, and stage 2 hypertension groups at all ages when compared with the normal BP group. However, the risk associated with the same CumBP exposure varied according to age group; for example, the risk associated with stage 1 hypertension decreased from 3.38 (95% CI: 1.54–7.43) at age <50 years to 1.58 (95% CI: 1.10–2.28) at age 69 years. The results were similar in the other BP groups and when CumBP was used as a continuous variable (Table 2). Although to our knowledge there are no previous studies similar to ours, we found that the results of subgroup analyses in two recent studies were generally consistent with our main outcome trend. Wang et al. (12) found that the risk of CVD associated with each standard deviation increase in CumSBP decreased from 1.69 (95% CI: 1.31–2.18) at age <45 years to 1.24 (95% CI: 1.11–1.36) at age 60 years. Reges et al. (10) showed that the risk of CVD associated with each standard deviation increase in CumSBP decreased from 1.42 (95% CI: 1.22–1.66) at the age of 45 years to 1.24 (95% CI: 1.16–1.33) at the age of >65 years. These results suggest that chronic exposure to high BP levels is more harmful in young and middle-aged individuals.

The 2017 ACC/AHA guidelines (17) recommend antihypertensive agents for primary prevention of CVD when BP reaches 130/80 mmHg, and the 10-year assessment of risk of ASCVD is ≥10%. Although, based on Supplementary Figure S3 and Table S4 displaying absolute risk, individuals in the elevated and stage-1 HT groups did not reach over 10% absolute risk over the study period. This might be explained due to the relatively short follow-up period. While the relative risk decreases as individuals age, the absolute risk increases. However, it is noteworthy that our study found that the risk of ASCVD in the group aged <50 years with CumBP in the elevated group (mean 123/77 mmHg) (Table 1) has increased by 238% (Table 2). Furthermore, our present findings are consistent with the results of a meta-analysis that included 17 studies with 4.5 million participants (22), which noted a 19% increase in the risk of CVD when baseline BP was 120–129/80–84 mmHg than when it was in the normal range (<120/80 mmHg). Considering that the present guidelines are based on single BP values, long-term cumulative BP could provide more comprehensive and accurate information. Therefore, clinicians should use CumBP values when evaluating and developing early interventions. Moreover, because the BP value when starting treatment in younger patients is based on the risk of ASCVD (≥10%), the window for optimal treatment may be missed in younger patients when using the current guidelines. Therefore, we recommend prompt intervention when CumBP is ≥120/80 mmHg. BP can be reduced to the desired level by improving diet and lifestyle, weight control, increasing physical activity, and using antihypertensive therapy to decrease the risk of ASCVD when the standard is still not reached.

Although we found that the risk of ASCVD in participants with stage 2 hypertension decreased in the different age groups after correction by antihypertensive medication, the risk decreased most rapidly in participants aged <50 years, with a decrease in the HR from 5.96 (95% CI: 2.92–12.16) before correction to 4.20 (95% CI: 1.91–9.25) after correction; and in older adults, the HR decreased from 2.67 (95% CI: 1.94–3.67) before correction to 2.44 (95% CI: 1.76–3.37) after correction (Table 2). These findings suggest that the risk of ASCVD is best minimized by administration of antihypertensive medication in young and middle-aged individuals (age <50 years) in the hypertensive population. The results reported by Li et al. (6) and those reported by Rahimi (23) are similar to our findings. Li et al. (6). noted that the benefit of early initiation of antihypertensive therapy was significantly greater in young and middle-aged individuals (age <49 years) than in older adults. This suggests that the benefit of antihypertensive medication in the hypertensive population varies according to age group. Thus, early detection, diagnosis, and treatment in young and middle-aged individuals (age <50 years) may maximize the benefit.

We found a cumulative effect on the ability of SBP to predict ASCVD events after replacing the SBP in the China-PAR risk prediction model with the 10-year TWA SBP (the C-statistic increased from 0.713 to 0.717; AUC increased from 0.718 to 0.723); furthermore, the NRI had an improved ability to predict ASCVD events (by 0.045). The TWA DBP improved its predictive power slightly (Table 3, Figure 2). This finding is similar to that in the study by Nwabuo et al. (24), who calculated CumBP for 15 years to assess the risk of CVD and found that CumSBP had an incremental effect and allowed reclassification of individual CVD risk (the C-statistic increased from 0.69 to 0.70 and NRI improved by 0.40). Although our study predicted a smaller improvement in risk, it still found a slight but statistically significant improvement in the ability to stratify the risk of ASCVD. Furthermore, considering the relative simplicity of BP measurement and monitoring and the ease of access to longitudinal electronic health record data, use of CumBP for prediction of ASCVD risk is feasible without changing standard clinical practice.

This study has several strengths. First, it explored for the first time the effect of CumBP on the risk of ASCVD according to age group in the large Kailuan study cohort using a window of up to 10 years. It emphasizes the importance of long-term maintenance of normal BP to minimize the risk of ASCVD and provides guidance on preventative strategies according to age group. Second, it used the BP grading criteria recommended by current guidelines, which are more convenient in terms of guiding physicians and patients when making decisions regarding management of BP. However, our study also has some limitations. First, our mean follow-up period of 4.08 years may not have been sufficient to fully accumulate the total number of ASCVD cases, which would have an impact on the stability of our results. However, the number of person-years of follow-up is sufficient to overcome this limitation. Second, the numbers of ASCVD cases within the different age groups were relatively small, which precluded our ability to analyze the data by type of ASCVD. Third, although we adjusted for risk factors for CVD in multivariate analysis, we could not exclude the effect of other confounding factors. Finally, in order to evaluate the 10-year period cumulative effect of BP, the exclusion of patients with newly diagnosed ASCVD within the 10-year period may lead to an underestimation of the cumulative effect of blood pressure on ASCVD risk.

In conclusion, 10 years of high CumBP exposure is a risk factor for ASCVD independent of and higher than the baseline BP level. This finding suggests that when focusing on single BP measurements, we should be aware of the harms associated with CumBP exposure, especially in individuals exposed to high CumBP levels at an early age (<50 years). Early intervention and antihypertensive treatment can achieve the greatest benefits in this population.

## Data Availability

The raw data supporting the conclusions of this article will be made available by the authors, without undue reservation.
