# Supplementary material for: Effect of 10-year cumulative blood pressure exposure on atherosclerotic cardiovascular disease of different age groups: kailuan cohort study
Source: Front Cardiovasc Med. 2024 Feb 1;11:1341097. doi: 10.3389/fcvm.2024.1341097 (PMC10867219; doi:10.3389/fcvm.2024.1341097)
Supplement: Supplementary file 1 [file Datasheet1.doc]

**Effect of 10-year Cumulative Blood Pressure Exposure on Atherosclerotic Cardiovascular Disease of Different Age Groups：Kailuan Cohort Study**

Lu Guo^#1^, Faming Tian^#1^, Jingyao Wang ^2^, Wenqi Xu^2^, Wenjuan Li^2^, Xiaoli Hou^1^, Mengyi, Zheng^3^, Xuemei Yang^2^ ,

Lishu Gao^4^, Shuohua Chen^5^, Nan Zhang^6*^, Shouling Wu^5*^.

^1^ the School of Public Health, North China University of Science and Technology. Tangshan, Hebei, China.

^2^the School of Clinical Medicine, North China University of Science and Technology. Tangshan, Hebei, China.

^3^Cardiovascular Center, Beijing Tongren Hospital, Capital Medical University, Beijing, China.

^4^ Department of Endocrinology, Tangshan People’s Hospital, Tangshan, Hebei, China.

^5^ Department of Cardiology, Kailuan General Hospital, Tangshan, Hebei, China.

^6^Department of orthopedics, Kailuan General Hospital, Tangshan, Hebei, China.

**Contributing authors:** guolu1013@163.com; tfm9911316@163.com;4387965@qq.com;xuwenqi15234007544@163.com;2069976478@qq.com;

houxiaoli1981@163.com;18331341285@163.com;yangxuemei_c@163.com;gaolishu2022@126.com;csh01062011@163.com;zhnan138@163.com; drwusl@163.com;

**Common first author:** Faming Tian, tfm9911316@163.com

#: Lu Guo and Faming Tian contributed equally to this work.

^*^**Corresponding author**: Shouling Wu, Department of Cardiology, Kailuan General Hospital, North China University of Science and Technology, No.57 Xinhua East Street, Tangshan, 063200, China. E-mail: drwusl@163.com

**Common corresponding author:** Nan Zhang, Department of orthopedics, Kailuan General Hospital, No.57 Xinhua East Street, Tangshan, 063200, China. E-mail: zhnan138@163.com

**Supplemental Materials**

**Supplementary table 1:** Covariates definitions.

**Supplementary table 2:** Calculation of TWA BP

**Supplementary table 3:** The distribution of endpoint events at different Time-Weighted Cumulative BP levels at different ages.

**Supplementary table 4:** Absolute risk, and sex-standardization absolute risk by different Time-averaged BP categories at different age groups.

**Supplementary table 5：**Risk of occurrence of Atherosclerotic Cardiovascular Disease events estimated based on Time-averaged BP at different ages sensitivity analysis.

**Supplementary table 6：**Risk of occurrence of Atherosclerotic Cardiovascular Disease events estimated based on Time-averaged BP at different ages sensitivity analysis.

**Supplementary Figure 1:** Flowchart of participants selection.

**Supplementary Figure 2:** Cumulative incidence of ASCVD in Time-averaged BP.

**Supplementary Figure 3.** Cumulative incidence of ASCVD in Time-averaged BP with different age.

**Supplementary table 1：**Covariates definitions.

| **Current-drinker** was defined as drinking for over 1 year, drinking on average ≥ 100 ml/d, and still drinking in the last year. |
| --- |
| **Current-smoker** was defined as having smoked at least one cigarette per day for at least one year. |
| **Body mass index** (BMI) was calculated as weight (kg) divided by the square of height (m^2^) and defined as obese at ≥28. |
| **Physical activity** was defined as exercise ≥3 times a week, with the duration of each exercise at least 30 min |
| **Education level** was defined as not attending school, elementary and junior high school education was defined as less than high school education, and secondary school and university undergraduate and college education were defined as high school and above. |
| **Diabetes** was defined as a fasting blood glucose level ≥7.0 mmol/L, current use of hypoglycemic medication despite a fasting blood glucose level <7.0 mmol/L, or a history of diabetes. |
| The severity of steatosis was differentiated by ultrasonography: mild (diffuse increase in fine echoes in liver parenchyma), moderate (diffuse increase in fine echoes with impaired visualization of the intrahepatic vessel borders and diaphragm), and severe (diffuse increase in fine echoes with non-visualization of the intrahepatic vessel borders and diaphragm). **Steatotic liver disease** was defined as steatosis (yes or no). |

**Supplementary table 2：**Calculation of TWA BP

| Taking participation in health checkups from 2006 to 2016 as an example: |
| --- |
| TWA BP = [(BP1 + BP2)/2 × time1-2 ] + [(BP2 + BP3)/2 × time2-3 ] +...+ [(BP5+ BP6)/2 × time5-6]/(time1-6)  where BP1, BP2, BP3, and BP6 are the BP levels calculated from BP values recorded during consecutive physical examinations, time1-2, time2-3, and time5-6 are the time intervals between two adjacent BP measurements, and time1-6 is the time interval between BP measurements obtained at the first (2006) and last (2016) physical examination. TWA BP for 2008–2018 was calculated in the same manner. |

**Supplementary Table 3.**The distribution of endpoint events at different Time-Weighted Cumulative BP levels at different ages

**P, comparison of adverse events among different BP groups.* *Statistically significant differences were assessed by the log-rank test.*

|  | Total | Normal BP | Elevated Blood Pressure | Stage 1 Hypertension | Stage 2 Hypertension | *P* value |
| --- | --- | --- | --- | --- | --- | --- |
| Total | 55,450 | 10,535 | 4,343 | 8,382 | 32,190 | - |
| ASCVD events, n (%) | 2197 (3.96) | 104 (0.99) | 105 (2.42) | 227 (2.71) | 1761 (5.47) | <0.01 |
| Ischemic Stroke, n (%) | 1357 (2.45) | 67 (0.64) | 59 (1.36) | 133 (1.59) | 1098 (3.41) | <0.01 |
| Myocardial infarction, n (%) | 698 (1.73) | 30 (0.28) | 37 (0.85) | 73 (0.87) | 558 (1.73) | <0.01 |
| Heart failure, n (%) | 235 (0.58) | 8 (0.08) | 11 (0.25) | 29 (0.35) | 187 (0.58) | <0.01 |
| <50 years | 14,338 | 4,953 | 1,356 | 2,587 | 5,442 | - |
| ASCVD events, n (%) | 137 (0.96) | 9 (0.18) | 9 (0.66) | 25 (0.97) | 94 (1.73) | <0.01 |
| 50-59 years | 16,245 | 3,068 | 1,125 | 2,491 | 9,561 | - |
| ASCVD events, n (%) | 600 (3.69) | 33 (1.08) | 27 (2.40) | 60 (2.41) | 480 (5.02) | <0.01 |
| 60-69 years | 18,677 | 2,142 | 1,439 | 2,660 | 12,436 | - |
| ASCVD events, n (%) | 946 (5.07) | 41 (1.91) | 43 (2.99) | 104 (3.91) | 758 (6.10) | <0.01 |
| ≥70 years | 6,190 | 372 | 423 | 644 | 4,751 | - |
| ASCVD events, n (%) | 514 (8.30) | 21 (5.65) | 26 (6.15) | 38 (5.90) | 429 (9.03) | <0.01 |

*Footnotes: ASCVD, Atherosclerotic cardiovascular disease.*

**Supplementary table 4.** Absolute risk, and sex-standardization absolute risk by different Time-averaged BP categories

at different age groups

| Age categories | Cumulative BP categories  (mmHg) | Cases/total  (N) | Absolute risk  (%) | Sex-standardization absolute risk  (95% CI) |
| --- | --- | --- | --- | --- |
| <50 years |  |  |  |  |
|  | Normal BP | 9/4953 | 0.18 | 0.19 (0.06-0.31) |
|  | Elevated Blood Pressure | 9/1356 | 0.66 | 0.62 (0.13-1.10) |
|  | Stage 1 Hypertension | 25/2587 | 0.97 | 0.96 (0.58-1.34) |
|  | Stage 2 Hypertension | 94/5442 | 1.73 | 1.73 (1.38-2.08) |
| 50-59 years |  |  |  |  |
|  | Normal BP | 33/3068 | 1.08 | 1.09 (0.72-1.46) |
|  | Elevated Blood Pressure | 27/1125 | 2.40 | 2.31 (1.42-3.21) |
|  | Stage 1 Hypertension | 60/2491 | 2.41 | 2.43 (1.81-3.05) |
|  | Stage 2 Hypertension | 480/9561 | 5.02 | 5.00 (4.55-5.45) |
| 60-69 years |  |  |  |  |
|  | Normal BP | 41/2142 | 1.91 | 1.92 (1.33-2.51) |
|  | Elevated Blood Pressure | 43/1439 | 2.99 | 2.96 (2.07-3.84) |
|  | Stage 1 Hypertension | 104/2660 | 3.25 | 3.18 (2.56-3.80) |
|  | Stage 2 Hypertension | 758/12436 | 6.10 | 6.11 (5.68-6.55) |
| ≥70 years |  |  |  |  |
|  | Normal BP | 21/372 | 6.18 | 6.17 (3.65-8.70) |
|  | Elevated Blood Pressure | 26/423 | 6.15 | 6.15 (3.78-8.51) |
|  | Stage 1 Hypertension | 38/644 | 5.90 | 5.79 (3.93-7.64) |
|  | Stage 2 Hypertension | 429/4751 | 9.03 | 9.04 (8.18-9.89) |

*Footnotes: SBP, systolic blood pressure; DBP, diastolic blood pressure; SD, standard deviation; 10y: ten years; TWA, Time-Weight average;*

*Adjusted absolute risk refers to absolute risk adjusted for sex.*

**Supplementary table 5.** Risk of occurrence of Atherosclerotic Cardiovascular Disease events estimated based on Time-averaged

BP at different ages sensitivity analysis (N=31,753)

| Age categories | Cumulative BP categories  (mmHg) | Cases/total  (N) | Incidence density  (per 1,000 person-years) | HR (95%CI) | | | |
| --- | --- | --- | --- | --- | --- | --- | --- |
|  |  |  |  | Model 1 | Model 2 | Model 3 | Model 4 |
| <50 years | Normal BP | 6/3368 | 0.45 | Reference | Reference | Reference | Reference |
|  | Elevated Blood Pressure | 8/937 | 2.23 | 4.16 (1.42-12.16) | 4.19 (1.43-12.27) | 4.23 (1.45-12.34) | 4.12 (1.40-12.08) |
|  | Stage 1 Hypertension | 19/1808 | 2.72 | 4.71 (1.84-12.04) | 4.76 (1.86-12.19) | 4.63 (1.81-11.82) | 4.51 (1.75-11.60) |
|  | Stage 2 Hypertension | 69/3860 | 4.58 | 6.98 (2.93-16.62) | 5.30 (2.04-13.80) | 5.11 (1.97-13.24) | 4.76 (1.75-12.96) |
|  | Per 1 SD (10yTWA-SBP) |  |  | 1.75 (1.43-2.14) | 1.60 (1.27-2.02) | 1.51 (1.18-1.92) | 1.50 (1.14-1.97) |
|  | Per 1 SD (10yTWA-DBP) |  |  | 1.67 (1.41-1.97) | 1.57 (1.30-1.89) | 1.47 (1.21-1.80) | 1.46 (1.17-1.81) |
| 50-59 years | Normal BP | 22/1958 | 2.88 | Reference | Reference | Reference | Reference |
|  | Elevated Blood Pressure | 14/702 | 5.09 | 1.54 (0.79-3.01) | 1.54 (0.79-3.01) | 1.56 (0.79-3.04) | 1.43 (0.73-2.81) |
|  | Stage 1 Hypertension | 38/1533 | 6.36 | 1.82 (1.08-3.09) | 1.82 (1.07-3.10) | 1.80 (1.06-3.05) | 1.64 (0.97-2.79) |
|  | Stage 2 Hypertension | 299/6163 | 12.68 | 3.40 (2.19-5.30) | 3.25 (2.02-5.21) | 2.99 (1.87-4.80) | 2.14 (1.29-3.53) |
|  | Per 1 SD (10yTWA-SBP) |  |  | 1.53 (1.38-1.69) | 1.47 (1.31-1.65) | 1.34 (1.19-1.51) | 1.17 (1.01-1.36) |
|  | Per 1 SD (10yTWA-DBP) |  |  | 1.43 (1.30-1.57) | 1.37 (1.24-1.53) | 1.27 (1.14-1.41) | 1.16 (1.03-1.32) |

Continued **supplementary table 5**

| Age categories | Cumulative BP categories  (mmHg) | Cases/total  (N) | Incidence density  (per 1,000 person-years) | HR (95%CI) | | | |
| --- | --- | --- | --- | --- | --- | --- | --- |
|  |  |  |  | Model 1 | Model 2 | Model 3 | Model 4 |
| 60-69 years | Normal BP | 16/977 | 4.25 | Reference | Reference | Reference | Reference |
|  | Elevated Blood Pressure | 11/692 | 4.06 | 0.91 (0.42-1.95) | 0.91 (0.42-1.96) | 0.85 (0.39-1.82) | 0.79 (0.36-1.70) |
|  | Stage 1 Hypertension | 38/1117 | 8.93 | 1.84 (1.02-3.30) | 1.84 (1.02-3.30) | 1.76 (0.98-3.17) | 1.62 (0.90-2.92) |
|  | Stage 2 Hypertension | 335/5730 | 15.63 | 3.02 (1.82-5.01) | 2.64 (1.57-4.43) | 2.36 (1.40-3.97) | 1.70 (0.98-2.95) |
|  | Per 1 SD (10yTWA-SBP) |  |  | 1.28 (1.18-1.40) | 1.20 (1.09-1.33) | 1.17 (1.06-1.30) | 1.02 (0.90-1.16) |
|  | Per 1 SD (10yTWA-DBP) |  |  | 1.21 (1.11-1.32) | 1.12 (1.02-1.24) | 1.11 (1.00-1.22) | 1.02 (0.91-1.14) |
| 70≥ years | Normal BP | 10/170 | 15.35 | Reference | Reference | Reference | Reference |
|  | Elevated Blood Pressure | 11/189 | 15.37 | 0.98 (0.42-2.33) | 1.00 (0.42-2.36) | 0.98 (0.41-2.31) | 0.98 (0.41-2.32) |
|  | Stage 1 Hypertension | 16/259 | 16.64 | 1.11 (0.50-2.47) | 1.12 (0.50-2.48) | 1.10 (0.50-2.44) | 1.10 (0.50-2.45) |
|  | Stage 2 Hypertension | 196/2290 | 23.19 | 1.44 (0.75-2.73) | 1.26 (0.65-2.45) | 1.24 (0.64-2.40) | 1.24 (0.61-2.53) |
|  | Per 1 SD (10yTWA-SBP) |  |  | 1.15 (1.02-1.29) | 1.10 (0.96-1.26) | 1.10 (0.96-1.26) | 1.12 (0.95-1.32) |
|  | Per 1 SD (10yTWA-DBP) |  |  | 1.00 (0.87-1.15) | 0.94 (0.81-1.09) | 0.95 (0.82-1.10) | 0.94 (0.80-1.09) |

*Footnotes: SBP, systolic blood pressure; DBP, diastolic blood pressure; SD, standard deviation; 10 y: ten years; TWA, Time-Weight average;*

*Model 1: adjusted for sex, drinking, smoking, education level, BMI, physical exercise, LDL-C, HDL-C, TG, FBG, steatotic liver disease, and*

*Antidiabetic treatment.*

*Model 2: adjusted for model 1 plus anti-hypertensive drug.*

*Model 3: adjusted for model 2 plus use of the Lipid-lowering drug.*

*Model 4: adjusted model 3 plus systolic blood pressure at baseline.*

**Supplementary table 6.** Risk of occurrence of Atherosclerotic Cardiovascular Disease events estimated based on Time-averaged

BP at different ages sensitivity analysis

| Age categories | Cumulative BP categories  (mmHg) | Cases/total  (N) | Incidence density  (per 1,000 person-years) | HR (95%CI) | | | |
| --- | --- | --- | --- | --- | --- | --- | --- |
|  |  |  |  | Model 1 | Model 2 | Model 3 | Model 4 |
| <50 years | Normal BP | 9/4953 | 0.47 | Reference | Reference | Reference | Reference |
|  | Elevated Blood Pressure | 9/1356 | 1.74 | 2.91 (1.14-7.41) | 2.94 (1.15-7.49) | 2.88 (1.13-7.34) | 2.67 (1.04-6.84) |
|  | Stage 1 Hypertension | 76/5846 | 3.34 | 4.75 (2.32-9.70) | 3.85 (1.84-8.05) | 3.70 (1.77-7.72) | 3.28 (1.54-6.97) |
|  | Stage 2 Hypertension | 43/2183 | 5.16 | 6.75 (3.19-14.29) | 4.83 (2.18-10.72) | 4.17 (1.87-9.30) | 3.37 (1.43-7.90) |
|  | Per 1 SD (10yTWA-SBP) |  |  | 1.84 (1.55-2.20) | 1.66 (1.35-2.03) | 1.53 (1.24-1.90) | 1.45 (1.12-1.87) |
|  | Per 1 SD (10yTWA-DBP) |  |  | 1.73 (1.50-1.99) | 1.60 (1.36-1.89) | 1.49 (1.26-1.77) | 1.44 (1.18-1.74) |
| 50-59 years | Normal BP | 33/3068 | 2.77 | Reference | Reference | Reference | Reference |
|  | Elevated Blood Pressure | 27/1125 | 6.09 | 1.95 (1.17-3.25) | 1.96 (1.18-3.26) | 1.99 (1.20-3.31) | 1.82 (1.09-3.03) |
|  | Stage 1 Hypertension | 289/7768 | 9.59 | 2.77 (1.93-4.00) | 2.56 (1.76-3.71) | 2.54 (1.75-3.68) | 2.01 (1.37-2.95) |
|  | Stage 2 Hypertension | 251/4284 | 15.57 | 4.32 (2.98-6.26) | 3.79 (2.57-5.59) | 3.49 (2.36-5.15) | 2.38 (1.57-3.61) |
|  | Per 1 SD (10yTWA-SBP) |  |  | 1.59 (1.46-1.72) | 1.54 (1.41-1.68) | 1.44 (1.31-1.57) | 1.26 (1.12-1.41) |

Continued **supplementary table 6.**

*Footnotes: SBP, systolic blood pressure; DBP, diastolic blood pressure; SD, standard deviation; 10 y: ten years; TWA, Time-Weight average;*

| Age categories  (years) | Cumulative BP categories  (mmHg) | Cases/total  (N) | Incidence density  (per 1,000 person-years) | HR (95%CI) | | | |
| --- | --- | --- | --- | --- | --- | --- | --- |
|  |  |  |  | Model 1 | Model 2 | Model 3 | Model 4 |
|  | Per 1 SD (10yTWA-DBP) |  |  | 1.47 (1.37-1.58) | 1.42 (1.31-1.54) | 1.34 (1.24-1.46) | 1.22 (1.11-1.34) |
| 60-69 years | Normal BP | 41/2142 | 4.97 | Reference | Reference | Reference | Reference |
|  | Elevated Blood Pressure | 43/1439 | 7.80 | 1.48 (0.96-2.26) | 1.48 (0.96-2.27) | 1.36 (0.89-2.09) | 1.27 (0.83-1.95) |
|  | Stage 1 Hypertension | 475/8974 | 14.07 | 2.37 (1.72-3.27) | 2.19 (1.58-3.03) | 2.00 (1.45-2.76) | 1.62 (1.16-2.27) |
|  | Stage 2 Hypertension | 387/6122 | 16.89 | 2.71 (1.95-3.75) | 2.38 (1.71-3.33) | 2.06 (1.47-2.87) | 1.51 (1.06-2.16) |
|  | Per 1 SD (10yTWA-SBP) |  |  | 1.33 (1.25-1.41) | 1.29 (1.21-1.38) | 1.24 (1.16-1.33) | 1.13 (1.03-1.23) |
|  | Per 1 SD (10yTWA-DBP) |  |  | 1.23 (1.16-1.31) | 1.18 (1.10-1.26) | 1.16 (1.08-1.24) | 1.08 (1.00-1.16) |
| 70≥ years | Normal BP | 21/372 | 14.98 | Reference | Reference | Reference | Reference |
|  | Elevated Blood Pressure | 26/423 | 17.11 | 1.11 (0.62-1.98) | 1.12 (0.63-1.99) | 1.11 (0.62-1.98) | 1.07 (0.60-1.90) |
|  | Stage 1 Hypertension | 271/3044 | 24.71 | 1.52 (0.97-2.38) | 1.38 (0.88-2.17) | 1.37 (0.87-2.15) | 1.19 (0.74-1.90) |
|  | Stage 2 Hypertension | 196/2351 | 23.15 | 1.42 (0.90-2.24) | 1.27 (0.80-2.01) | 1.26 (0.80-2.00) | 1.05 (0.95-1.72) |
|  | Per 1 SD (10yTWA-SBP) |  |  | 1.15 (1.06-1.24) | 1.11 (1.02-1.21) | 1.11 (1.01-1.21) | 1.06 (0.95-1.18) |
|  | Per 1 SD (10yTWA-DBP) |  |  | 1.05 (0.96-1.15) | 1.01 (0.92-1.11) | 1.02 (0.93-1.12) | 0.98 (0.89-1.08) |

*Model 1: adjusted for sex, drinking, smoking, education level, BMI, physical exercise, LDL-C, HDL-C, TG, FBG, steatotic liver disease, and*

*Antidiabetic treatment.*

*Model 2: adjusted for model 1 plus anti-hypertensive drug.*

*Model 3: adjusted for model 2 plus use of the Lipid-lowering drug.*

*Model 4: adjusted model 3 plus systolic blood pressure at baseline.*


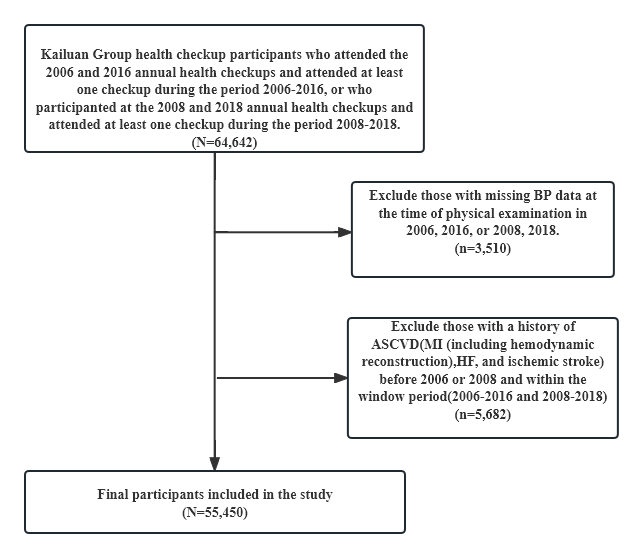


**Supplementary Figure 1.** Flowchart of participants selection

**
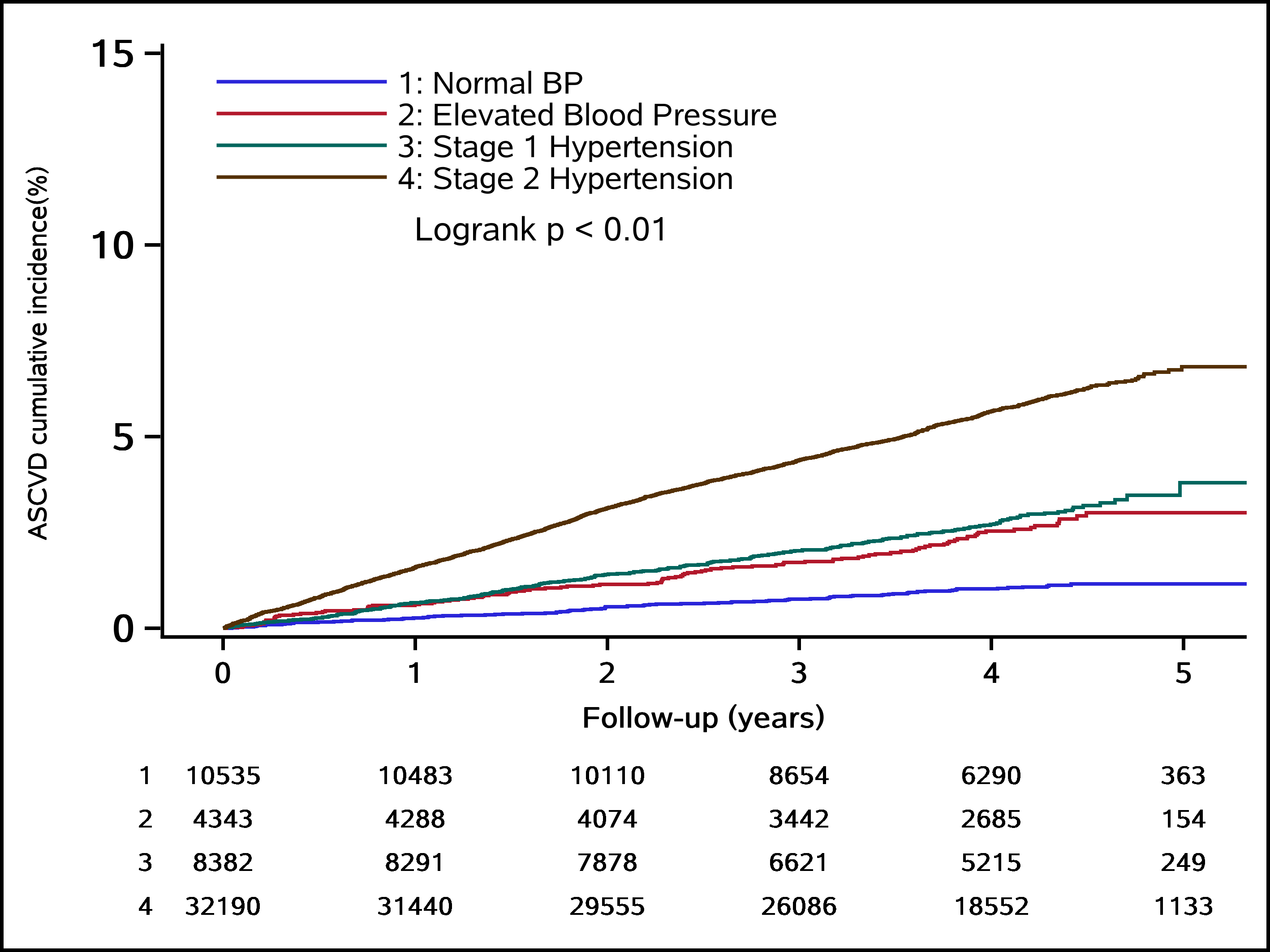
**

**Supplementary Figure 2.** Cumulative incidence of ASCVD in Time-averaged BP


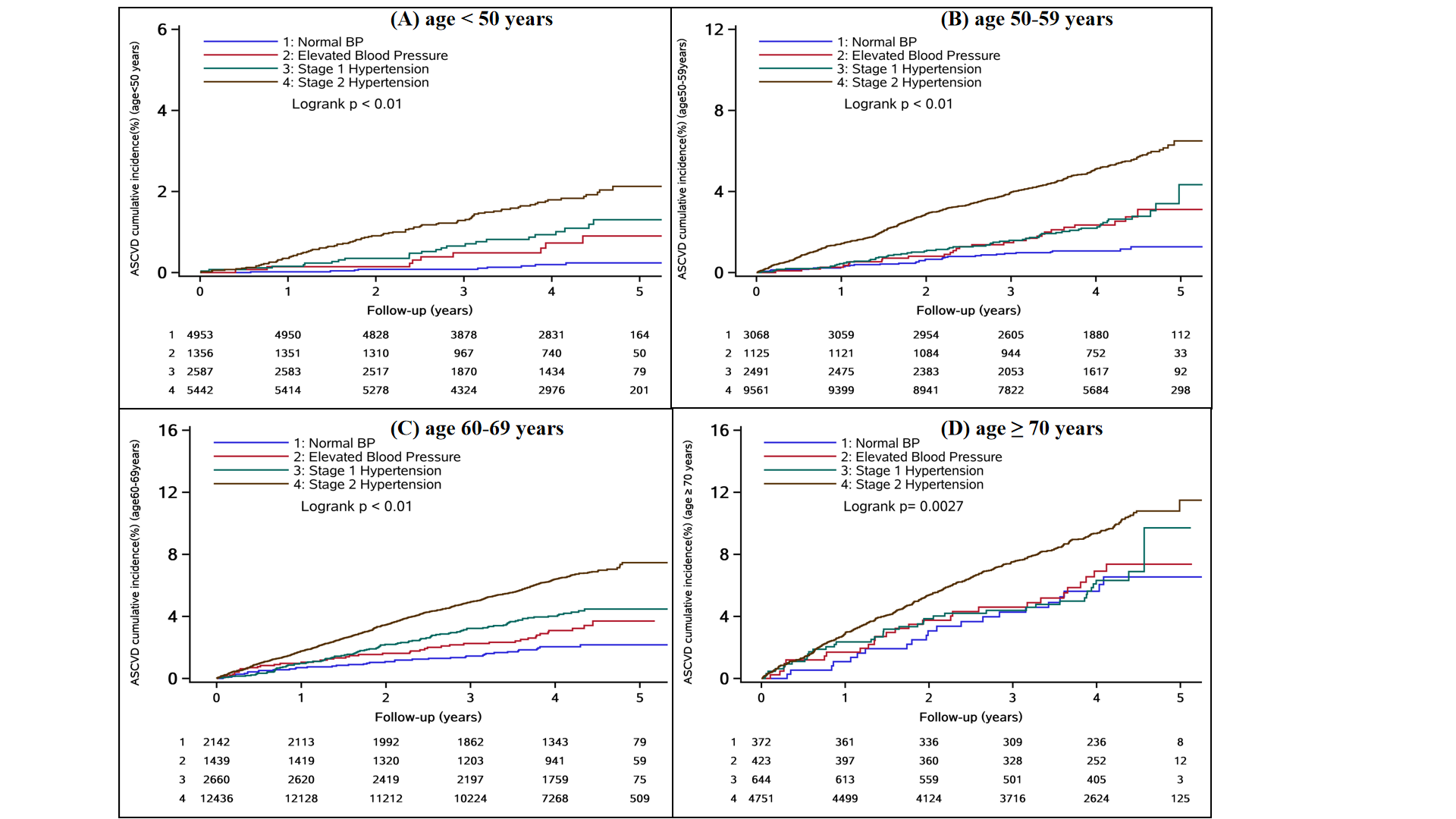


**Supplementary Figure 3.** Cumulative incidence of ASCVD in Time-averaged BP with different age.
